# The Effects of Filter’s Class, Cutoff Frequencies, and Independent Component Analysis on the Amplitude of Somatosensory Evoked Potentials Recorded from Healthy Volunteers

**DOI:** 10.3390/s19112610

**Published:** 2019-06-08

**Authors:** Muhammad Samran Navid, Imran Khan Niazi, Dina Lelic, Asbjørn Mohr Drewes, Heidi Haavik

**Affiliations:** 1 Mech-Sense, Department of Gastroenterology and Hepatology, Aalborg University Hospital, 9000 Aalborg, Denmark; m.navid@rn.dk (M.S.N.); dilelic@gmail.com (D.L.); amd@rn.dk (A.M.D.); 2 Department of Clinical Medicine, Aalborg University, 9000 Aalborg, Denmark; 3 Centre for Chiropractic Research, New Zealand College of Chiropractic, 1060 Auckland, New Zealand; heidi.haavik@nzchiro.co.nz; 4 Faculty of Health & Environmental Sciences, Health & Rehabilitation Research Institute, AUT University, 0627 Auckland, New Zealand; 5 Centre for Sensory-Motor Interactions (SMI), Department of Health Science and Technology, Aalborg University, 9220 Aalborg, Denmark

**Keywords:** EEG, preprocessing, SEPs, filtering, ICA

## Abstract

Objective: The aim of this study was to investigate the effects of different preprocessing parameters on the amplitude of median nerve somatosensory evoked potentials (SEPs). Methods: Different combinations of two classes of filters (Finite Impulse Response (FIR) and Infinite Impulse Response (IIR)), three cutoff frequency bands (0.5–1000 Hz, 3–1000 Hz, and 30–1000 Hz), and independent component analysis (ICA) were used to preprocess SEPs recorded from 17 healthy volunteers who participated in two sessions of 1000 stimulations of the right median nerve. N30 amplitude was calculated from frontally placed electrode (F3). Results: The epochs classified as artifacts from SEPs filtered with FIR compared to those filtered with IIR were 1% more using automatic and 140% more using semi-automatic methods (both *p* < 0.001). There were no differences in N30 amplitudes between FIR and IIR filtered SEPs. The N30 amplitude was significantly lower for SEPs filtered with 30–1000 Hz compared to the bandpass frequencies 0.5–1000 Hz and 3–1000 Hz. The N30 amplitude was significantly reduced when SEPs were cleaned with ICA compared to the SEPs from which non-brain components were not removed using ICA. Conclusion: This study suggests that the preprocessing of SEPs should be done carefully and the neuroscience community should come to a consensus regarding SEP preprocessing guidelines, as the preprocessing parameters can affect the outcomes that may influence the interpretations of results, replicability, and comparison of different studies.

## 1. Introduction

Somatosensory evoked potentials (SEPs) are elicited by stimulating the peripheral nerve at a distal site, e.g., the median nerve at the wrist [1]. SEPs are widely-utilized and have been used as an intraoperative monitoring method for more than 30 years [2]. SEPs are also used to understand the effect of different treatments and drugs on the central nervous system (CNS), and the level of the CNS where the changes occur [3].

To effectively and efficiently analyze SEPs, an improvement of the signal-to-noise ratio of the EEG signals is required. This is achieved by using filters. Filtering is the most basic and major part of the preprocessing of electroencephalography (EEG) data. There are two classes of digital filters, Finite Impulse Response (FIR) and Infinite Impulse Response (IIR). The difference between the two is that IIR uses some of the filter’s output as input, making it recursive in nature. IIR is computationally faster but less stable compared to its counterpart, FIR. The output signal is dependent on different parameters, such as the filter type, filter order, and cutoff frequencies. Filter type (low-pass, high-pass, band-pass, and band-stop) along with cutoff frequencies define which parts of the signal spectrum are to be kept and which are to be removed. The order of the filter defines the roll-off or transition bandwidth, with a higher order making ‘sharper’ filters with steeper roll-offs. For more details about signal processing, filter theory and design, we would like to recommend the freely available book found in [4] and paper [5]. Due to the nature of the process itself, filtering may seriously modify the appearance of signals, and thereby affect the results obtained. Filtering can introduce artificial components [6] and distortions in the onset latency [7] and amplitude of the brain processes [5].

Various guidelines have been published to standardize the recording, processing, utilization, and interpretation of SEPs [1,8,9,10,11]. However, in spite of different guidelines on preprocessing SEPs, and the literature [5,6,12,13] with suggestions on filtering EEG, especially evoked–related potential (ERP) data, there are inconsistencies in the use and reporting of filtering in the studies. We searched PubMed for articles, published between 2000 and 2017, which used SEPs and found that there is no consensus on the class and order of filters used, their cutoff frequencies, and their reporting. Even the articles that used median nerve stimulation to evoke somatosensory potentials published by the co-authors of this paper used filters with different properties, e.g., [14] used 1–70 Hz, 24 dB/octave, [15] used 1–1000 Hz with no mention of the filter’s roll-off, whereas [16,17,18] used 3–1000 Hz, 6 dB/octave. None of these mentioned the class of the filter used. This shows the variation in the use and reporting of filters in the literature.

Therefore, the objective of this study was to investigate the effects of the class of filter used, the cutoff frequencies, and the use of independent component analysis (ICA) on the amplitudes of SEPs evoked by stimulating the median nerve. For this purpose, we used two classes of filters, Kaiser-windowed (FIR) and Butterworth (IIR), with three bandpass frequencies: 0.5–1000 Hz, 3–1000 Hz, and 30–1000 Hz, processed with or without ICA, which was used to remove non-brain components from the data. The choice of filters and cutoff frequencies is based on the previous usage and recommendations of analyzing ERP and SEP data obtained from the literature search.

## 2. Methods

The study was conducted according to the Declaration of Helsinki. The Northern A Health and Disability Ethics Committee of Auckland (approval number: 14NTA232) approved the study. The data were recorded at the Centre for Chiropractic Research, New Zealand College of Chiropractic, Auckland, New Zealand.

### 2.1. Experimental Protocol

The subjects participated in two sessions, separated by at least a week. Each session consisted of median nerve stimulation and EEG recording. During each session, the subjects were seated comfortably in the supine position and were asked to keep their eyes open. A secondary person blinded the data analyst by assigning random numbers to each dataset making it impossible to identify and associate data with particular sessions and subjects during analysis. The data analyst was unblinded when statistics was to be performed.

### 2.2. Subjects

Seventeen healthy subjects (9 males, 27.3 ± 5.6 years) participated in the study. The subjects gave their written informed consent to participate in the study.

### 2.3. Median Nerve Stimulation

The median nerve was stimulated by applying electrical pulses at the right wrist through the stimulation electrodes (Neuroline 700, AMBU A/S, Denmark) connected to the electrical stimulator (Digitimer DS7AH, UK) to evoke somatosensory potentials (SEPs). The stimulation pulse was monophasic, with a width of 0.2 ms and a frequency of 2.3 Hz. A total of 1000 pulses were given at the motor threshold of each subject, which was defined as the lowest intensity that elicited a visible twitch of the thumb.

### 2.4. EEG

The EEG was recorded from 62 channels according to the international 10–20 electrode system (Klem et al., 1999) using the REFA amplifier (TMSi, Twente, The Netherlands) at a sampling rate of 2048 Hz. The ground electrode was placed at AFz. The impedance was kept below 10 kΩ. The subjects were asked to keep eye blinks, eye movements, and facial movements to the minimum.

The EEG preprocessing was performed offline using EEGLAB version 14.1.1 [19] and ERPLAB version 6.1.4 [20] running on MATLAB 2015b (The MathWorks, Inc., Natick, MA, USA.). Custom scripts were developed in MATLAB utilizing EEGLAB, ERPLAB and MATLAB functions to perform the analysis.

The raw EEG was imported into MATLAB using EEGLAB. The EEG was truncated to contain data from the 30 s preceding the first stimulation to 30 s following the last stimulation. The PREP pipeline version 0.55.1 [21] was used to identify noisy channels, remove the line noise, and average reference the data.

**Figure 1:**
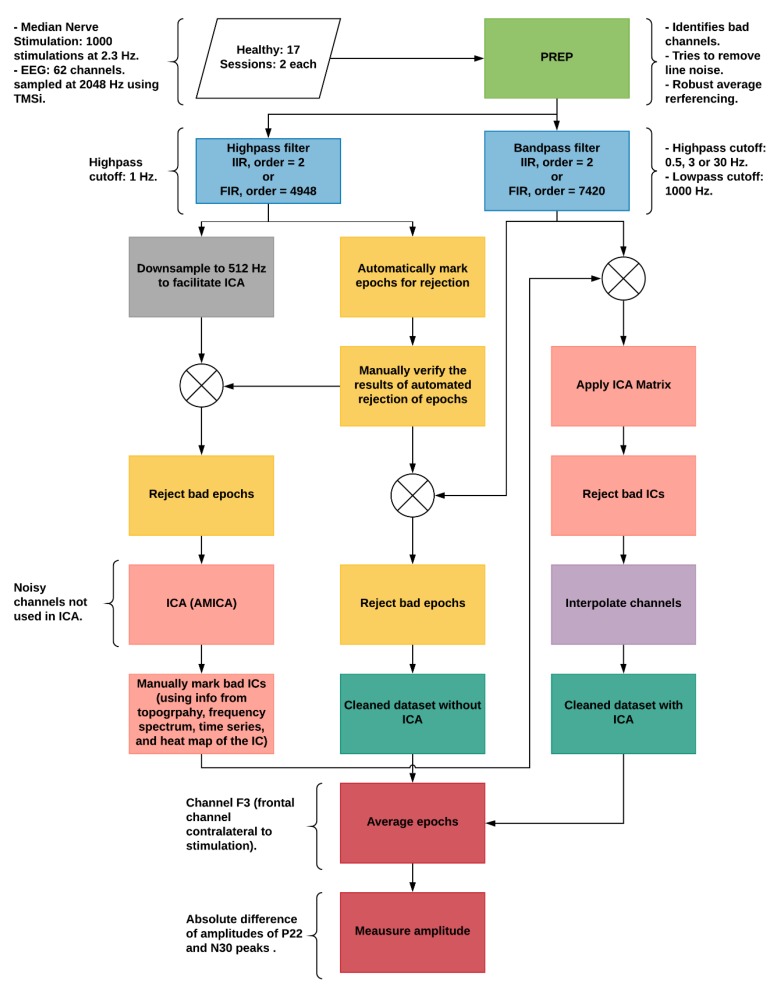
Methodology overview. The colors group similar processes or sub-processes. Blue is filter properties, yellow corresponds to artifact detection and rejection, salmon pink represents steps related to independent component analysis (ICA), dark green are cleaned datasets, and red are related to somatosensory evoked potential (SEP) averaging and amplitude. Abbreviations: FIR = Finite Impulse Response; IIR = Infinite Impulse Response; AMICA = adaptive mixture ICA; IC = Independent Component.

In the following sections, the finite impulse response (FIR) was performed using EEGLAB’s function *pop_firws*, whereas infinite impulse response (IIR) filtering was done using MATLAB’s functions *butter* and *filtfilt*. The order of FIR was either 4948 which corresponded to the transition bandwidth of 1.5 Hz or 7420 which corresponded to the transition bandwidth of 1 Hz. The FIR window used in both cases was Kaiser, with a *β* of 5.653. The IIR filter used was the 2nd order Butterworth filter. The epochs were always extracted from −100 to 150 ms with respect to stimulus and baseline corrected using the pre-stimulus time period. Figure 1 shows the overview of the EEG processing pipeline.

#### 2.4.1. Artifact Identification

For identifying and marking the epochs contaminated with artifacts, the continuous PREPed EEG was high-pass filtered with a cutoff frequency of 1 Hz, with either a FIR (order = 4948) or an IIR filter. The filtered data was segmented into epochs.

An epoch was marked as artifact epoch using ERPLAB if any of the EEG channels in that epoch possessed one or more of the following properties: (i) absolute voltage above 100 μV, (ii) peak-to-peak voltage of more than 150 μV in a sliding window of size 200 ms with a step size of 100 ms, (iii) voltage greater than 100 μV resulting from step-function with a sliding window of size 200 ms and a step size of 50 ms, (iv) sample-to-sample difference of more than 50 μV, or (v) absolute voltage less than 2 μV for more than 125 ms. The time corresponding to the stimulus artifact (−2 to 2 ms) was excluded in all checks. Afterward, all the epochs were manually checked for correctness of automatic detection of artifacts. If any epoch was incorrectly marked, or was not marked but was an artifact, it was unmarked or marked, respectively. The epochs that had step-like artifacts in the frontal channels were not removed as they corresponded to eye-blinks and eye-movements [12]. It is to be noted that the bad epochs were not rejected but only marked as artifacts at this stage.

#### 2.4.2. ICA

Independent component analysis (ICA) decomposes EEG data into components that are maximally independent temporally (i.e., spatially fixed and temporally distinct) [22]. In this study, the adaptive mixture ICA (AMICA) algorithm was used to decompose EEG data into independent components (ICs), since it has been shown that the performance of AMICA is superior to that of other ICA algorithms [23].

The continuous 1 Hz high-pass filtered EEG (from Section 2.4.1) was downsampled to 512 Hz, epoched, and the epochs previously identified as artifacts were rejected. The EEG channels identified as noisy channels by the PREP pipeline were removed. The resulting EEG was decomposed into ICs using AMICA.

The ICA weights obtained were applied to the band-pass filtered with bad epochs removed PREPed data. The cutoff frequency of band-pass filter was 0.5–1000 Hz and the filter class was same as the high-pass filter class used in artifact identification. The FIR filter order was 7420. All the ICs from these datasets were manually classified either as a brain component or a non-brain component, corresponding to muscle, channel or line noise, or eye activity. The ICs were categorized using their spatial distributions (scalp topographies), time courses, spectrograms, event-related potential (ERP) images, and equivalent current dipole models using the guidelines from [24,25] and the website, https://labeling.ucsd.edu/.

#### 2.4.3. Cleaned Datasets

The zero-phase band-pass filtering was performed on PREPed data using one of the two classes of filters: FIR (order = 7420) or IIR, with one of the three cutoff frequencies: 0.5–1000 Hz; 3–1000 Hz; or 30–1000 Hz.

The band-pass filtered data were segmented into epochs. These six datasets are referred to ‘filtered and no ICA’ in the following text. Afterward, the ICA weights obtained in Section 2.4.2 from the analogous filter class were applied to each of these datasets, and the ICs marked as non-brain components were removed, resulting in six datasets, which are referred to as ‘filtered and ICA’ in the subsequent text.

### 2.5. N30 Amplitude

The good epochs were averaged and the amplitude of the N30 peak was calculated from channel F3 [15], contralateral to the stimulated nerve. The most positive and the most negative peaks were identified automatically in the windows from 15–25 ms and 25–35 ms, respectively. The identified peaks were manually verified and modified by an expert if (i) they were out of this time period or (ii) there was more than one peak in a window, which lead to the wrong identification of the peak. The N30 amplitude was taken as the absolute difference of the amplitudes of these two peaks.

### 2.6. Statistics

The data are presented as a mean ± SD unless otherwise indicated. The statistical significance threshold was set at *p* < 0.05. R version 3.5.1 [26] was used for all statistical procedures.

Dependent t-tests were performed to find the difference in the number of epochs rejected automatically and semi-automatically from FIR and IIR filtered data.

The linear mixed effect model (LMM) was used to identify the effects of filter class, cutoff frequencies and use of ICA, and their interactions on the N30 amplitude. The between-subject variance was estimated using random intercept in the model. The model used was:

(1) $$\begin{array}{ll} N30\_Amplitude_{i,j} & \\ & =\beta_{0}+\beta_{1}*FILTER_{i,j}+\beta_{2}*ICA_{i,j}+\beta_{3}*FREQUENCY1_{i,j}+\beta_{4}\\ & *\;FREQUENCY2_{i,j}+\beta_{5}*FILTER_{i,j}*ICA_{i,j}+\beta_{6}*FILTER_{i,j}\\ & *\;FREQUENCY1_{i,j_{1}}+\beta_{7}*FILTER_{i,j}*FREQUENCY2_{i,j}+\beta_{8}*ICA_{i,j}\\ & *\;FREQUENCY1_{i,j}+\beta_{9}*ICA_{i,j}*FREQUENCY2_{i,j}+\beta_{10}*FILTER_{i,j}\\ & *\;ICA_{i,j}*FREQUENCY1_{i,j}+\beta_{11}*FILTER_{i,j}*ICA_{i,j}\\ & *\;FREQUENCY2_{i,j}+z_{i}+\varepsilon_{i,j} \end{array}$$

where i is the subject number, j is the measurement number, filter is FIR when FILTER=0 and IIR when FILTER=1, ICA is used when ICA=1 and not used when ICA=0, cutoff frequency is 3–1000 Hz when FREQUENCY1=1 and FREQUENCY2=0, 30–1000 Hz when FREQUENCY1=0 and FREQUENCY2=1, and 0.5–1000 Hz when FREQUENCY1=FREQUENCY2=0. The model was implemented using lme4 package version 1.1.18.1 [27] in R using the syntax:

(2) N30_Amplitude~filter∗isICA∗frequency+(1|subject)

Since the data were not normally distributed and had unequal variances, we used gamma distribution to model the data. The choice of the link function (identity or log) was evaluated using Akaike information criterion corrected for small samples (AICc). The AICc penalizes both under fitting and over fitting. We used the log link. The contrasts were obtained using the emmeans package version 1.2.4 [28], adjusted for multiple comparisons using Tukey’s HSD.

## 3. Results

All subjects successfully completed the experiments. Data from all of them were included for analysis.

### 3.1. Number of Artifacts

Using the automated settings for identifying epochs contaminated with artifacts, there were more epochs marked for rejection when data were filtered with FIR (229.59 ± 257.83) compared to IIR (227.29 ± 258.39), t(33) = 7.23, *p* < 0.0001, *r* = 0.99, 95% CI = [1.65, 2.94]. Similarly, with semi-automated marking of artifacts, on average there were more artifacts rejected from FIR filtered data (54.50 ± 56.37) compared to IIR filtered data (22.71 ± 22.43), t(33) = 3.91, *p* = 0.0004, *r* = 0.57, 95% CI = [15.24, 48.35].

### 3.2. N30 Amplitude

**Figure 2:**
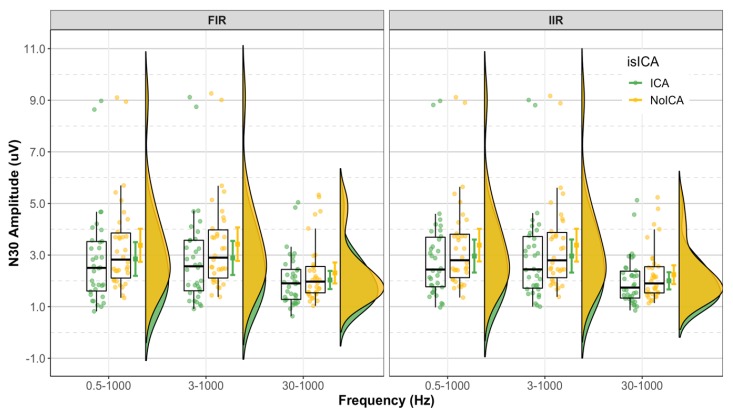
N30 amplitude. Dots represent N30 amplitude of each dataset. Boxplots show the median, 25th and 75th percentiles. The error bars represent mean ± 95% CI. The distribution plots show the density distribution estimated by a Gaussian kernel with SD of 1.5. The figure was made using the code provided by [29].

**Figure 3:**
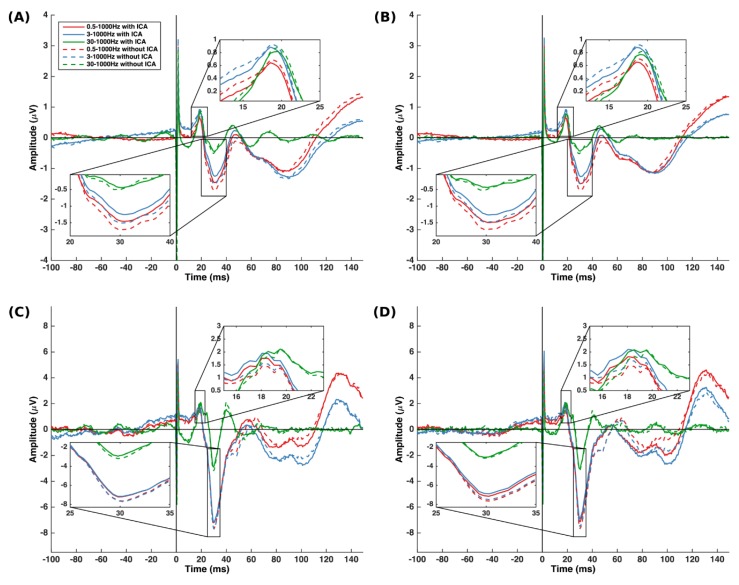
SEPs. Grand average SEPs filtered with (**A**) FIR and (**B**) IIR. Mean SEPs from one session of a representative participant processed with (**C**) FIR and (**D**) IIR filter.

The LMM showed no significant interactions between the filter class, cutoff frequencies, and use of ICA. The main effects of cutoff frequencies (*p* < 0.001) and use of ICA (*p* < 0.001) were significant. Table 1 presents the mean N30 amplitude, and Figure 2 shows the distribution of the N30 amplitude in the 12 groups. Figure 3 shows the grand average N30 amplitude and the mean of epochs preprocessed with FIR and IIR with different cutoff frequencies and ICA from a representative subject. The intercept and slopes from the model are given in Table 2. The estimated N30 amplitude obtained using the emmeans function in R is given in Table 3.

#### 3.2.1. Effect of Filter Class

**Figure 4:**
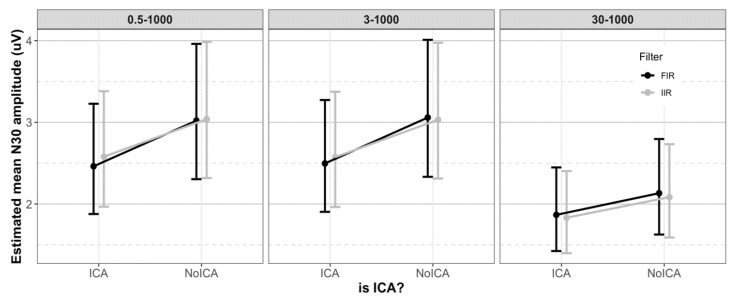
The effect of filter class. The error bar shows estimated mean N30 amplitude ± 95% CI. The class of filter (FIR or IIR) had no effect on the N30 amplitude.

There were statistically no significant differences between N30 amplitudes filtered with FIR or IIR, irrespective of the cutoff frequencies and use of ICA. Figure 4 shows the effect of filter class on N30 amplitude, and contrasts are given in Table 4.

#### 3.2.2. Effect of Cutoff Frequency

**Figure 5:**
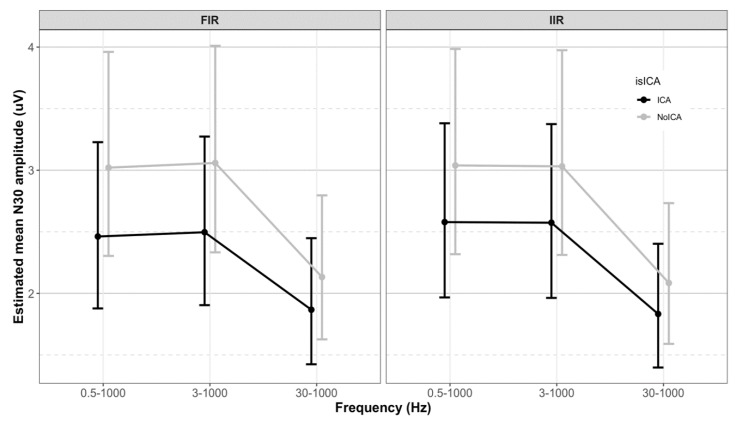
The effect of cutoff frequency and the use of ICA. The error bar shows estimated mean N30 amplitude ± 95% CI. The 30–1000 Hz band showed significantly lower N30 amplitude compared to the 0.5–1000 Hz and 3–1000 Hz bands. The use of ICA significantly reduced the N30 amplitude.

The N30 amplitudes filtered with frequency bands 0.5–1000 Hz and 3–1000 Hz were similar. However, filtering with the 30–1000 Hz band significantly lowered the N30 amplitude compared to the 0.5–1000 Hz (*p* < 0.0001) and 3–1000 Hz (*p* < 0.0001) filtered data. Figure 5 shows the effect of cutoff frequencies on N30 amplitude. The contrasts obtained using the emmeans function in R are given in Table 5.

#### 3.2.3. Effect of ICA

The use of ICA reduced the N30 amplitude significantly for all combinations of filter class and frequency bands (30–1000 Hz: *p* < 0.004, rest: *p* < 0.001). Figure 5 shows the effect of ICA on N30 amplitude. The contrasts obtained using the emmeans function in R are given in Table 6.

## 4. Discussion

In this study, we investigated the effects of filter class, cutoff frequencies, and the use of ICA on the amplitudes of somatosensory potentials evoked by stimulating the median nerve. We found that there were more epochs classified as artifacts from EEG filtered with FIR compared to those filtered with IIR using automatic and semi-automatic methods. We found a reduced N30 amplitude when EEG was cleaned with ICA compared to the EEG from which non-brain components were not removed using ICA. Compared to the bandpass frequencies 0.5–1000 Hz and 3–1000 Hz, we found lower amplitude for N30 when the EEG was filtered with the bandpass frequency of 30–1000 Hz. There were no substantial differences in N30 amplitudes between FIR and IIR filtered EEG.

### 4.1. Selection of Preprocessing Parameters

We found inconsistencies in the usage and reporting of the filter cutoff frequency bands in the literature. Therefore, we selected three frequency bands to evaluate the effects on the N30 amplitude when EEG is preprocessed with them. We selected the 0.5–1000 Hz based on the guidelines recommended for processing EEG and ERP [5,12], whereas the 3–1000 Hz band was selected, as it was one of the most reported. The 30–1000 Hz band was selected, as it is the recommended frequency band for SEP analysis [1,8,9,10]. Table A1 contains a brief literature review.

The filter class IIR (Butterworth) was chosen, as it was the most commonly used, whereas FIR was selected, as it is recommended for EEG preprocessing [5,30].

ICA was used, as it can be utilized to remove artifacts and non-brain components from the EEG data, which can be beneficial for patients’ data or data recorded in noisy environments.

### 4.2. Number of Artifacts

The parameters used to classify epochs contaminated with artifacts were obtained by randomly selecting eight datasets and adjusting the parameters by visual inspection of the results of the automatic classification of epochs as artifacts. There were significant differences in the number of epochs classified as artifacts between FIR and IIR filtered data. However, since there can be significant variability in the size and shape of the artifacts across subjects, this one-size-fits-all approach may not be optimal. It was observed that the automated settings marked all epochs as artifacts in a few datasets.

The semi-automated validation of epochs as artifacts, which was done by the person blind to the dataset and filter class, showed significant effects of the filter class on the N30 amplitude, likely due to the difference in the number of epochs identified as artifacts.

Keeping the filter class constant, the number of epochs stays same and shouldn’t affect the results of the effects of the cutoff frequencies and the use of ICA, as it can be treated as a within-subject manipulation. However, comparison of N30 amplitude filtered with FIR and that filtered with IIR in combination with either the bandpass frequency or the use of ICA can be biased, since the number of epochs used for averaging was different and quantifying the N30 amplitude using the peak amplitudes is affected by the number of trials used for averaging [12].

The effect of manual classification can be reduced by using artifact rejection parameters set for each individual subject, as suggested by [12].

### 4.3. Filter Class

The IIR (Butterworth) filtered EEG showed a similar N30 amplitude compared to FIR (Kaiser window) filtered EEG, irrespective of the cutoff frequencies and the use of ICA to remove non-brain components from the data.

IIR filtering is the most commonly used in the electrophysiological studies. The likely reason for this is the that it has been used since older times, when computers were not as fast as today, and the practice continues. However, with modern computers, the computational cost of using FIR is slightly higher compared to the IIR filters. FIR filtering is recommended for offline analysis by [5,30], as FIR filters are more stable and less likely to produce phase distortions. Keeping the phase information correct is important in phase-connectivity analysis.

We found it easier to design the FIR filter compared to the IIR filter by specifying the cutoff frequencies and the transition bandwidths in Hz and getting the order of the filter as a result, which was used for filtering the data. For the IIR, the filter coefficients are obtained by specifying the cutoff frequency and the filter order. The transition bandwidth is not a straightforward result and needs the interpretation of the impulse response. In this way, we felt we had more control over the design of the FIR filter.

### 4.4. Frequency Band

The recommendation for recording and analyzing SEPs by [1,8,9,10] suggested using the 30–1000 Hz band. However, we found inconsistencies in the following of these guidelines in the literature. Therefore, we used three different passbands, 0.5–1000 Hz, 3–1000 Hz and 30–1000 Hz to assess the effect of filter’s cutoff frequencies on the N30 amplitude.

The 30–1000 Hz band always showed a lower N30 amplitude. The possible reason is that all the lower frequency spectra from EEG are removed, whereas the EEG follows the 1/f function, which means that the power at lower frequencies has a larger magnitude compared to the power at higher frequencies [30]. The distribution of the N30 amplitude data showed more outliers compared to the other two frequency bands when ICA was not used to remove the non-brain components from the EEG. The likely reason is that the muscle activity, which has a high frequency, was still present in the data, and since average referencing was used, the muscle activity might also have affected the N30 amplitude.

The N30 amplitude with the 3–1000 Hz band was similar to that of the 0.5–1000 Hz band. However, using a high-pass cutoff of 0.5 Hz removes the DC offset and slow drifts, but keeps the lower spectrum of the EEG.

A sharper transition is suggested for the high-pass filter (to get the intended lower spectrum), and a shallower transition for the low-pass filter (to avoid distortion and spread of the signal in time-domain), which makes filtering EEG using the successive application of a high-pass filter and a low-pass filter, instead of a single bandpass filter with a similar transition at both ends [5].

### 4.5. ICA

The N30 amplitude was found to be lower when ICA was used and non-brain components were removed. Since the EEG components corresponding to the muscle, channel or line noise, or eye activity were removed, the overall amplitude of the N30 was reduced.

Use of ICA helps to keep more trials in the data, as the trials contaminated with eye blinks and eye movements can be corrected instead of being rejected. The channels contaminated with muscle activity or environmental noise can be fixed by removing the corresponding component, reducing the need for interpolation of the channel. Lastly, the line noise and its harmonics are removed more efficiently with ICA without distorting the signals, as the notch filter usually used to remove the mains noise, produce strong artifacts [12]. 

### 4.6. Limitations

Due to the small sample size, it is possible that the results from the LMM are biased. We, therefore, also analyzed the data using a three-way repeated measures ANOVA. The detailed procedure and results are given in the Supplementary Materials. A brief summary of the differences of results obtained from LMM and ANOVA is given here.

With respect to the model, the difference between the LMM and ANOVA was that the two-way interactions of filter and frequency, and the use of ICA and frequency in ANOVA were significant.

The Tukey-Kramer test revealed that for FIR and ICA, the N30 amplitudes filtered with frequency bands 0.5–1000 Hz and 3–1000 Hz were significantly different, but this was not the case for LMM contrasts. The other difference was that according to Tukey-Kramer test, the N30 amplitude was not affected by the use of ICA (either with FIR or IIR) when the cutoff frequency of 30–1000 Hz was used for filtering, whereas LMM contrasts showed that use of ICA significantly reduced N30 amplitude for all combinations of filter class and frequency bands.

One limitation of the current study is that the significant differences in amplitudes under varying techniques does not show which method is statistically more efficient, i.e., which technique is best able to differentiate between the different treatments or populations. This result should be incorporated in future work.

### 4.7. Toolboxes

Currently, there are many toolboxes for analyzing the neural data, e.g., EEGLAB [19], Fieldtrip [31], and ERPLAB [20]. These toolboxes have made analyzing EEG easier. However, they act as a black box if their functionalities and the default functions and parameters are not carefully understood before utilizing them for the analysis of EEG data. There are considerable differences among them regarding how filtering is performed. The default filter class of EEGLAB is FIR, whereas Fieldtrip and ERPLAB use the Butterworth filter. In Fieldtrip, the default order of the Butterworth high-pass and low-pass filters is, 6 whereas it is 4 for the bandpass and bandstop. The transition bandwidth for the FIR filter cannot be specified with ERPLAB, but it is possible to do that with EEGLAB. Additionally, EEGLAB, by default, keeps the data in single precision, but filtering should always be performed on the double precision data (EEGLAB converts the data to double precision when filtering but returns the result in single precision if EEGLAB’s default memory options are used). [5] showed how different toolboxes returned different outputs despite having the same filter parameters as inputs. Therefore, we would like to recommend that it is important to understand how different analysis are implemented in the toolboxes and what the default parameters are. Manually setting the filter parameters is recommended.

### 4.8. Recommendations

Based on the results of this study, and recommendations from other studies (e.g., [5,12,30]), for replication and comparison of different studies, we would like to recommend that

1. In the preprocessing section of the methodology, filter class (FIR, IIR, high-pass, low-pass, band-pass), order, slope/transition bandwidth, cutoff frequencies should be reported.
2. Filtering should always be performed on continuous data in double precision.
3. Use FIR, as it is more stable and less likely to introduce phase distortions. Additionally, we found it easier to design and understand the FIR response giving the cutoff frequencies and transition bandwidth in Hz, instead of roll-off in octaves or decades.
4. Use 0.5 Hz (or lower) as the cutoff frequency for the high-pass filter to remove DC and slow drifts but keeps the rest of the EEG spectra.
5. Use ICA to remove non-brain/noisy components from the EEG, as this may improve the statistical power by keeping a higher number of trials in the data and maintaining the brain activity for the analysis.

## 5. Conclusions

We found a reduced N30 amplitude when the EEG was cleaned with ICA compared to the EEG from which non-brain components were not removed using ICA. Compared to the bandpass frequencies, 0.5–1000 Hz and 3–1000 Hz, we found a lower amplitude of N30 when the EEG was filtered with the bandpass frequency of 30–1000 Hz. We found no substantial differences in N30 amplitudes between FIR and IIR filtered EEG. Considering the effects of the class of filter, the cutoff frequencies and the use of ICA, it is recommended to be careful when selecting the preprocessing parameters, as they can affect the outcomes, which may be relevant not only to studies based on SEPs but also to laser-evoked potentials and other ERPs, which are being used in clinical research and applications, as they may affect the interpretations of results, replicability, and comparison of studies.

## Figures and Tables

**Table 1:** N30 amplitude.

Filter	Frequency (Hz)	ICA	N30 Amplitude (μV) (Mean ± SD)
FIR	0.5–1000	Yes	2.85 ± 1.87
		No	3.38 ± 1.84
	3–1000	Yes	2.89 ± 1.89
		No	3.42 ± 1.85
	30–1000	Yes	2.03 ± 1.01
		No	2.31 ± 1.17
IIR	0.5–1000	Yes	2.96 ± 1.83
		No	3.39 ± 1.81
	3–1000	Yes	2.96 ± 1.84
		No	3.38 ± 1.82
	30–1000	Yes	2.00 ± 0.96
		No	2.24 ± 1.04

**Table 2:** Estimated coefficients from the statistical model. Significant effects (*p* < 0.05) are in bold text.

Model Coefficients		Estimate	Standard Error	*t* Value	*p* Value
$\beta_{0}$	Intercept	1.11	0.14	8.00	**<0.0001**
$\beta_{1}$	Filter = IIR	0.01	0.04	0.13	0.8930
$\beta_{2}$	ICA = Yes	−0.21	0.04	−4.59	**<0.0001**
$\beta_{3}$	Frequency = 3–1000	0.01	0.04	0.28	0.7800
$\beta_{4}$	Frequency = 30–1000	−0.35	0.04	−7.82	**<0.0001**
$\beta_{5}$	Filter = IIR: ICA = Yes	0.04	0.06	0.64	0.5210
$\beta_{6}$	Filter = IIR: Frequency = 3–1000	0.02	0.06	−0.24	0.8120
$\beta_{7}$	Filter = IIR: Frequency = 30–1000	0.03	0.06	−0.46	0.6480
$\beta_{8}$	ICA = Yes: Frequency = 3–1000	0.00	0.06	0.03	0.9800
$\beta_{9}$	ICA = Yes: Frequency = 30–1000	0.07	0.06	1.14	0.2540
$\beta_{10}$	Filter = IIR: ICA = Yes: Frequency = 3–1000	−0.00	0.09	−0.01	0.9910
$\beta_{11}$	Filter = IIR: ICA = Yes: Frequency = 30–1000	−0.04	0.09	−0.41	0.6830

**Table 3:** Estimated N30 amplitude from the statistical model. Abbreviations: LCL = Lower Confidence Level; UCL = Upper Confidence Level.

Filter	Frequency (Hz)	ICA	N30 Amplitude (μV)	Standard Error (μV)	95% CI LCL	95% CI UCL
FIR	0.5–1000	Yes	2.46	0.34	1.88	3.23
		No	3.02	0.42	2.30	3.96
	3–1000	Yes	2.50	0.35	1.90	3.27
		No	3.06	0.42	2.33	4.01
	30–1000	Yes	1.87	0.26	1.42	2.45
		No	2.13	0.29	1.63	2.80
IIR	0.5–1000	Yes	2.58	0.36	1.97	3.38
		No	3.04	0.42	2.32	3.98
	3–1000	Yes	2.57	0.36	1.96	3.37
		No	3.03	0.42	2.31	3.97
	30–1000	Yes	1.83	0.25	1.40	2.40
		No	2.08	0.29	1.59	2.73

**Table 4:** Estimated contrasts of filter class (FIR/IIR).

ICA	Frequency (Hz)	Ratio (FIR/IIR)	Standard Error (μV)	95% CI LCL	95% CI UCL	*z* Ratio	*p* Value
Yes	0.5–1000	0.95	0.04	0.87	1.04	−1.04	0.2972
	3–1000	0.97	0.04	0.89	1.06	−0.68	0.4943
	30–1000	1.02	0.05	0.93	1.11	0.42	0.6755
No	0.5–1000	0.99	0.04	0.91	1.08	−0.13	0.8932
	3–1000	1.01	0.04	0.92	1.10	0.20	0.8396
	30–1000	1.02	0.05	0.94	1.12	0.51	0.6095

**Table 5:** Estimated contrasts of frequency bands (Hz) (0.5–1000/3–1000, 0.5–1000/30–1000 and 3–1000/30–1000). Significant effects (*p* < 0.05) are in bold text.

Filter	ICA	Contrast	Ratio	Standard Error (μV)	95% CI LCL	95% CI UCL	*z* Ratio	*p* Value
FIR	Yes	0.5–1000/3–1000	0.99	0.04	0.89	1.09	−0.32	0.9467
		0.5–1000/30–1000	1.32	0.06	1.19	1.46	6.20	**<0.0001**
		3–1000/30–1000	1.34	0.06	1.20	1.48	6.52	**<0.0001**
	No	0.5–1000/3–1000	0.99	0.04	0.89	1.10	−0.28	0.9579
		0.5–1000/30–1000	1.42	0.06	1.28	1.57	7.82	**<0.0001**
		3–1000/30–1000	1.43	0.06	1.29	1.59	8.09	**<0.0001**
IIR	Yes	0.5–1000/3–1000	1.00	0.04	0.90	1.11	0.04	0.9989
		0.5–1000/30–1000	1.41	0.06	1.27	1.56	7.66	**<0.0001**
		3–1000/30–1000	1.40	0.06	1.27	1.56	7.62	**<0.0001**
	No	0.5–1000/3–1000	1.00	0.04	0.90	1.11	0.06	0.9982
		0.5–1000/30–1000	1.46	0.06	1.31	1.62	8.46	**<0.0001**
		3–1000/30–1000	1.45	0.06	1.31	1.61	8.41	**<0.0001**

**Table 6:** Estimated contrasts of ICA (ICA/No ICA). Significant effects (*p* < 0.05) are in bold text.

Filter	Frequency (Hz)	Ratio (NoICA/ICA)	Standard Error (μV)	95% CI LCL	95% CI UCL	*z* Ratio	*p* Value
FIR	0.5–1000	1.23	0.05	1.12	1.34	4.59	**<0.0001**
	3–1000	1.23	0.05	1.12	1.34	4.56	**<0.0001**
	30–1000	1.14	0.05	1.05	1.25	2.98	**0.0029**
IIR	0.5–1000	1.18	0.05	1.08	1.29	3.69	**0.0002**
	3–1000	1.18	0.05	1.08	1.29	3.67	**0.0002**
	30–1000	1.14	0.05	1.04	1.24	2.89	**0.0039**

## Supplementary Materials

The following are available online at https://www.mdpi.com/1424-8220/19/11/2610/s1, Table S1. ANOVA table. Significant effects (*p* < 0.05) are in bold text, Table S2. Pairwise comparisons by filter class. Significant effects (*p* < 0.05) are in bold text, Table S3. Pairwise comparisons by frequency. Significant effects (*p* < 0.05) are in bold text, Table S4. Pairwise comparisons by use of ICA. Significant effects (*p* < 0.05) are in bold text, Figure S1. The effect of filter class. The error bar shows mean N30 amplitude ± 95% CI. The class of filter (FIR or IIR) had no effect on the N30 amplitude, Figure S2. The effect of cutoff frequency and the use of ICA. The error bar shows mean N30 amplitude ± 95% CI. The 30–1000 Hz band showed significantly lower N30 amplitude compared to the 0.5–1000 Hz and 3–1000 Hz bands. The use of ICA significantly reduced the N30 amplitude except when filtered with 30–1000 Hz.

## Appendix A

The literature search was conducted using PubMed. The search terms used were one or a combination of several terms from the following: electroencephalography, EEG, event-related potentials, ERP, somatosensory-evoked potentials, SEPs, SSEPs, preprocessing, data analysis, artifact rejection, and noise. Furthermore, the inclusion criteria included that the studies were conducted on adult individuals and published in English during the period between 2000 and 2017. A brief literature review, which involved median nerve stimulation, is given in Table A1.

**Table A1:** Brief literature review.

Study	Electrodes ^1^	SEP Components	Filter Class	Filter Order	Filter Roll Off/Transition Bandwidth	Filter Cutoff Frequency (Hz)
(Di Lorenzo et al., 2016) [32]	Skin	N9, N13, N20, P25, N33	−	−	−	0–450
(Puta et al., 2016) [33]	Skin	N9, N20	−	−	−12 dB/octave	3–750
(Haavik-Taylor and Murphy, 2007) [34]	Skin	N9, N11, N13, P14-N18 complex, N20 (P14-N20, N20-P27 complexes), N30 (P22-N30 complex)	−	−	−6 dB/octave	3–1000
(Fedele et al., 2017) [35]	ECoG	N20, HFO	Butterworth	2	−12 dB/octave	N20 (30–1000), HFO (400–1000)
(Roser et al., 2016) [36]	ECoG	N20, P40	−	−	−	10–1000
(Baars and von Klitzing, 2017) [37]	Skin	N20	−	−	−	3–800
(Ares et al., 2018) [38]	Scalp needle and Skin	−	−	−	−	Scalp needle (3–300), Skin (30–1000)
(Burnos et al., 2016) [39]	Skin and ECoG	N20, HFO	Butterworth	2	−12 dB/octave	N20 (30–1000), HFO (500–1000)
(Sakuma et al., 2004) [40]	Skin	N9, N13, N20, P25, HFO	−	−	−	HFO (400–800), rest (0.3–3000)
(Bailey et al., 2016) [41]	Skin	N20-P25 complex	−	−	−	2–2500
(Maegaki et al., 2000) [42]	ECoG	N20, P20, P25, N30	−	−	−	30–2000
(Endisch et al., 2016) [43]	Skin	N20, N20-P25 complex, HFO	HFO (FIR)	−	−	HFO (450–750), rest (5–1500)
(Murakami et al., 2008) [44]	Skin	P14–N20, N20–P25, P25–N33, baseline-N13, N13onset-N13peak complexes	−	−	−	0.3–3000
(Andrew et al., 2015) [45]	Skin	N9, N13, N18 (P14–N18 complex), N20 (P14–N20 complex), N24 (P22–N24 complex), N30 (P22–N30 complex)	−	−	−	0.2–1000
(Mideksa et al., 2012) [46]	Skin	N20	−	−	−	0.01–200
(Han et al., 2014) [47]	Skin	N20, P37	−	−	−	20–3000
(Boostani et al., 2016) [48]	Skin	N9, N11, N13, N20	−	−	−	5–2000
(Lelic et al., 2016) [15]	Skin	N30	−	−	−	1–1000
(Haavik et al., 2017) [16]	Skin	N9, N11, N13, P14, N18, N20, N30	−	−	−6 dB/octave	3–1000
(Taylor and Murphy, 2010) [18]	Skin	N9, N11, N13, P14, N18, N20, N30	−	−	−6 dB/octave	3–1000
(Tinazzi et al., 2000) [49]	Skin	N9, N13, P14, N20, P27, N30	−	−	−6 dB/octave	5–1500
(Haavik Taylor and Murphy, 2007) [17]	Skin	N9, N11, N13, P14, N18, N20, N30	−	−	−6 dB/octave	3–1000
(Adhikari et al., 2016) [50]	Skin	N20, P24, P40, N48	−	−	−	10–250
(Balzamo et al., 2004) [51]	ECoG	N20-P30, P20-N30	−	−	−	1–1000
(Hoshiyama and Kakigi, 2000) [52]	Skin	N20, P25, N33, P20, N30	−	−	−	1–500
(Kaňovský et al., 2003) [53]	ECoG	N30	−	−	−	10–1000
(Tinazzi et al., 2000) [54]	Skin	N13, P14, N20, P27, N30	−	−	−6 dB/octave	5–1500
(Taylor and Murphy, 2010) [55]	Skin	N9, N11 N13, P14, N18, N20 N30	−	−	−	3–1000
(Van Rijn et al., 2009) [56]	Skin	N9, N14, N20, N35	−	−	−	30–1000
(Costa et al., 2007) [57]	Scalp needle	−	−	−	−	30–1000
(Jin et al., 2014) [58]	Scalp needle	N20, P25	−	−	−	30–1000

^1^ Skin electrodes include scalp EEG electrodes and any other electrodes placed on skin, e.g., at Erb’s point.
