# Supplementary material for: The Effects of Filter’s Class, Cutoff Frequencies, and Independent Component Analysis on the Amplitude of Somatosensory Evoked Potentials Recorded from Healthy Volunteers
Source: Sensors (Basel). 2019 Jun 8;19(11):2610. doi: 10.3390/s19112610 (PMC6603557; doi:10.3390/s19112610)
Supplement: Supplementary file 1 [file sensors-19-02610-s001.pdf]

The Effects of Filter's Class, Cutoff Frequencies, and Independent Component Analysis on the Amplitude of Somatosensory Evoked Potentials Recorded from Healthy Volunteers

Muhammad Samran Navid [1,2,3] Imran Khan Niazi [3,4,5,*], Dina Lelic [1], Asbjørn Mohr Drewes [1,2] and Heidi Haavik [3]

[1] Mech-Sense, Department of Gastroenterology and Hepatology, Aalborg University Hospital, 9000 Aalborg, Denmark; m.navid@rn.dk (M.S.N.); dilelic@gmail.com (D.L.); amd@rn.dk (A.M.D.)

[2] Department of Clinical Medicine, Aalborg University, 9000 Aalborg, Denmark

[3] Centre for Chiropractic Research, New Zealand College of Chiropractic, 1060 Auckland, New Zealand; heidi.haavik@nzchiro.co.nz

[4] Faculty of Health & Environmental Sciences, Health & Rehabilitation Research Institute, AUT University, 0627, Auckland, New Zealand

[5] Centre for Sensory-Motor Interactions (SMI), Department of Health Science and Technology, 9220 Aalborg University, Aalborg, Denmark

* Correspondence: imran.niazi@nzchiro.co.nz

1 Method

1.1 Statistics

The data are presented as mean ± SD unless otherwise indicated. The statistical significance threshold was set at $p < 0.05$. MATLAB 2015b (The MathWorks, Inc., Natick, MA, USA.). was used for the statistical procedures.

A three-way repeated measures analysis of variance (ANOVA) with factors filter class (FIR and IIR), use of ICA (yes and no), and cutoff frequency (0.5-1000Hz, 3-1000Hz, and 30-1000Hz) was performed to identify the effects of different preprocessing parameters on the N30 amplitude. Tukey-Kramer test was performed in order to do pairwise comparisons.

2 Results

For ease of reading, meaningful pairwise comparisons are split into three tables.

2.1 ANOVA

The Mauchly's test indicated that the assumption of sphericity had been violated for the main effects of frequency, $W = 0.005$, $p < 0.0001$, $e = 0.50$, and the two-way interactions of filter and frequency, $W = 0.086$, $p < 0.0001$, $e = 0.52$, and use of ICA and frequency, $W = 0.056$, $p < 0.0001$, $e = 0.51$, and the three-way interaction of filter, user of ICA and frequency, $W = 0.061$, $p < 0.0001$, $e = 0.52$. Therefore, the degrees of freedom were corrected using Greenhouse-Geisser estimates of sphericity.

Table S1 contains the results obtained for the repeated measures ANOVA. There were significant main effects of the use of ICA and cutoff frequency, and significant interactions of filter and cutoff frequency, and the use of ICA and cutoff frequency.

Table S1. ANOVA table. Significant effects ($p < 0.05$) are in bold text.

Predictor	df_num	df_den	Epsilon	SS_num	SS_den	F	*p* value	ges
(Intercept)	1.00	33.00		3238.95	924.49	115.62	**0.000**	0.76
filter	1.00	33.00		0.01	2.53	0.11	0.738	0.00
isICA	1.00	33.00		16.61	35.24	15.55	**0.000**	0.02
filter x isICA	1.00	33.00		0.18	3.50	1.72	0.199	0.00

frequency	1.00	33.08	0.50	92.18	74.73	40.71	**0.000**	0.08
filter x frequency	1.04	34.47	0.52	0.21	1.03	6.71	**0.013**	0.00
isICA x frequency	1.03	33.94	0.51	1.12	1.05	35.12	**0.000**	0.00
filter x isICA x frequency	1.03	34.04	0.52	0.03	0.32	3.42	0.072	0.00

Note. df_num indicates degrees of freedom numerator. df_den indicates degrees of freedom denominator. Epsilon indicates Greenhouse-Geisser multiplier for degrees of freedom, p-values and degrees of freedom in the table incorporate this correction. SS_num indicates sum of squares numerator. SS_den indicates sum of squares denominator. ges indicates generalized eta-squared.

2.2 Effect of filter class

There were statistically no significant differences between N30 amplitudes filtered with FIR or IIR, irrespective of the cutoff frequencies and use of ICA (all $p > 0.05$). Table S2 contains the results of the pairwise comparisons by filter class. Figure S1 shows the effect of filter class on N30 amplitude.

Table S2. Pairwise comparisons by filter class. Significant effects ($p < 0.05$) are in bold text.

Pair 1	Pair 2	*p* value	95% CI LCL	95% CI UCL
FIR_ICA_0.5-1000	IIR_ICA_0.5-1000	0.7885	-0.33	0.10
FIR_ICA_3-1000	IIR_ICA_3-1000	0.9846	-0.29	0.14
FIR_ICA_30-1000	IIR_ICA_30-1000	1.0000	-0.16	0.23
FIR_NoICA_0.5-1000	IIR_NoICA_0.5-1000	1.0000	-0.06	0.05
FIR_NoICA_3-1000	IIR_NoICA_3-1000	0.3348	-0.01	0.09
FIR_NoICA_30-1000	IIR_NoICA_30-1000	0.9351	-0.09	0.22

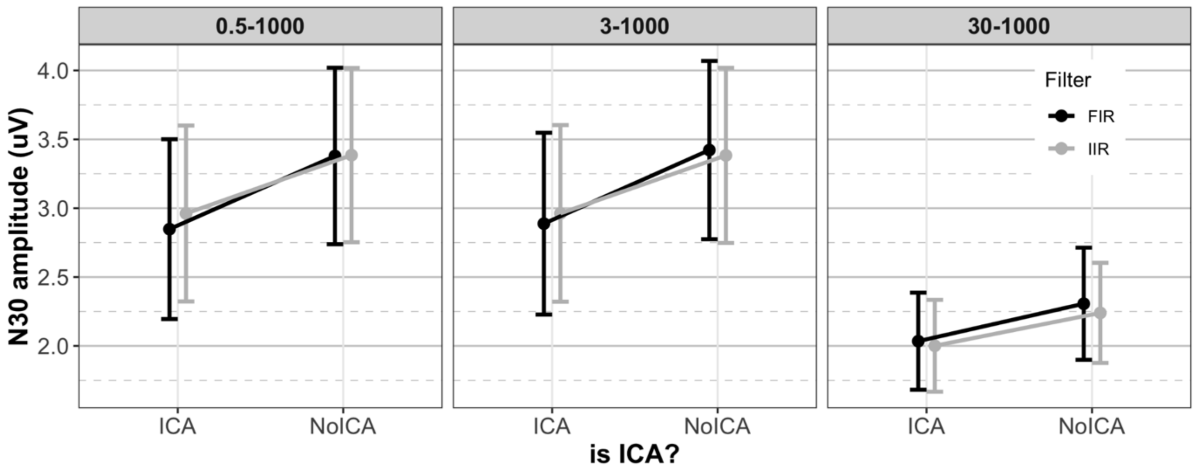

Figure S1. The effect of filter class. The error bar shows mean N30 amplitude ±95% CI. The class of filter (FIR or IIR) had no effect on the N30 amplitude.

2.3 Effect of cutoff frequency

The pairwise comparisons showed that with FIR, whether the data was cleaned with ICA or not, the N30 amplitude was lowest for the data filtered with 30-1000Hz and highest for the data filtered with 3-1000Hz (all $p < 0.05$). For IIR, the pairwise comparisons showed whether the data was cleaned with ICA or not, the N30 amplitude was similar in the data filtered with 0.5-1000Hz and 3-1000Hz (both $p > 0.05$), however, it was lower when filtered with 30-1000Hz (all $p < 0.0001$) compared to 0.5-1000Hz and 3-1000Hz filtered data. Table S3 contains the results of the pairwise comparisons by the filter's cutoff frequencies. Figure S2 shows the effect of cutoff frequencies on N30 amplitude.

Table S3. Pairwise comparisons by frequency. Significant effects ($p < 0.05$) are in bold text.

Pair 1	Pair 2	*p* value	95% CI LCL	95% CI UCL
FIR_ICA_0.5-1000	FIR_ICA_3-1000	**0.0173**	-0.08	0.00
FIR_ICA_0.5-1000	FIR_ICA_30-1000	**0.0019**	0.21	1.41
FIR_ICA_3-1000	FIR_ICA_30-1000	**0.0011**	0.25	1.46
IIR_ICA_0.5-1000	IIR_ICA_3-1000	1.0000	-0.02	0.02
IIR_ICA_0.5-1000	IIR_ICA_30-1000	**0.0001**	0.39	1.53
IIR_ICA_3-1000	IIR_ICA_30-1000	**0.0001**	0.39	1.53

FIR_NoICA_0.5-1000	FIR_NoICA_3-1000	**0.0006**	-0.07	-0.01
FIR_NoICA_0.5-1000	FIR_NoICA_30-1000	**0.0000**	0.53	1.61
FIR_NoICA_3-1000	FIR_NoICA_30-1000	**0.0000**	0.56	1.67
IIR_NoICA_0.5-1000	IIR_NoICA_3-1000	1.0000	-0.02	0.03
IIR_NoICA_0.5-1000	IIR_NoICA_30-1000	**0.0000**	0.61	1.68
IIR_NoICA_3-1000	IIR_NoICA_30-1000	**0.0000**	0.61	1.68

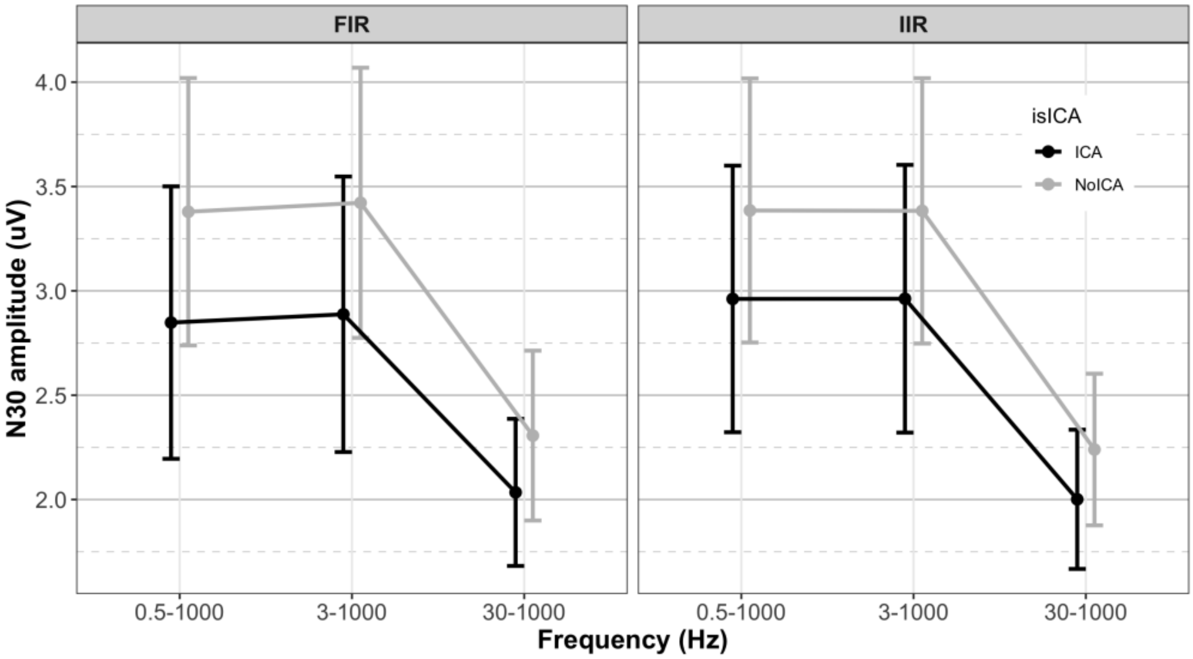

Figure S2. The effect of cutoff frequency and the use of ICA. The error bar shows mean N30 amplitude ±95% CI. The 30-1000Hz band showed significantly lower N30 amplitude compared to the 0.5-1000Hz and 3-1000Hz bands. The use of ICA significantly reduced the N30 amplitude except when filtered with 30-1000Hz.

2.4 Effect of ICA

The pairwise comparisons showed that the N30 amplitude was not affected by the use of ICA (either with FIR or IIR) when the cutoff frequency of 30-1000Hz was used for filtering (both $p > 0.05$). However, the N30 amplitude was lower when ICA was used (either with FIR or IIR) with the cutoff frequencies of 0.5-1000Hz and 3-1000Hz (all $p < 0.05$). Table S4 contains the results of the pairwise comparisons by the use of ICA. Figure S2 shows the effect of ICA on N30 amplitude.

Table S4. Pairwise comparisons by use of ICA. Significant effects ($p < 0.05$) are in bold text.

Pair 1	Pair 2	*p* value	95% CI LCL	95% CI UCL
FIR_ICA_0.5-1000	FIR_NoICA_0.5-1000	**0.0185**	-1.01	-0.05
FIR_ICA_3-1000	FIR_NoICA_3-1000	**0.0146**	-1.00	-0.07
FIR_ICA_30-1000	FIR_NoICA_30-1000	0.4971	-0.69	0.14
IIR_ICA_0.5-1000	IIR_NoICA_0.5-1000	**0.0026**	-0.74	-0.10
IIR_ICA_3-1000	IIR_NoICA_3-1000	**0.0020**	-0.73	-0.11
IIR_ICA_30-1000	IIR_NoICA_30-1000	0.0756	-0.49	0.01
